# Consequences of screening in cervical cancer: development and dimensionality of a questionnaire

**DOI:** 10.1186/s40359-018-0251-2

**Published:** 2018-08-10

**Authors:** John Brodersen, Volkert Siersma, Hanne Thorsen

**Affiliations:** 1 Section of General Practice and Research Unit for General Practice, Department of Public Health, Faculty of Health Sciences, University of Copenhagen, Øster Farimagsgade 5, P. O. Box 2099, DK-1014 Copenhagen, Denmark; 2 Primary Health Care Research Unit, Region Zealand, Denmark

**Keywords:** Cervical cancer, Psychometrics, Public health, Questionnaire development, Secondary prevention

## Abstract

**Background:**

Cervical cancer screening will inevitably lead to unintentional harmful effects e.g. detection of indolent pathological conditions defined as overdetection or overdiagnosis. Overdiagnosis often leads to overutilisation, overtreatment, labelling and thereby negative psychosocial consequences. There is a lack of adequate psychosocial measures when it comes to measurement of the harms of medical screening. However, the Consequences of Screening questionnaire (COS) has been found relevant and comprehensive with adequate psychometric properties in breast and lung cancer screening. Therefore, the aim of the present study was to extend the Consequences of Screening Questionnaire for use in cervical cancer screening by testing for content coverage, dimensionality, and reliability.

**Methods:**

In interviews, the suitability, content coverage, and relevance of the COS were tested on participants in cervical screening. The results were thematically analysed to identify the key consequences of abnormal screening results. Item Response Theory and Classical Test Theory were used to analyse data. Dimensionality, invariance, and reliability were established by item analysis, examining the fit between item responses and Rasch models.

**Results:**

All COS items were found relevant by the interviewees and the ten COS constructs were confirmed each to be unidimensional in the Rasch models. Ten new themes specifically relevant for participants having abnormal cervical screening result were extracted from the interviews: ‘Uncertainty about the screening result’, ‘Uncertainty about future pregnancy’, ‘Change in body perception’, ‘Change in perception of own age’, ‘Guilt’, ‘Fear and powerlessness’, ‘Negative experiences from the pelvic examination’, ‘Negative experiences from the examination’, ‘Emotional reactions’ and ‘Sexuality’ Altogether, 50 new items were generated: 10 were single items. Most of the remaining 40 items were confirmed to fit Rasch models measuring ten different constructs. However, the two items in the scale ‘Change in perception of own age’ both possessed differential item functioning in relation to time, which can bias longitudinal repeated measurement.

**Conclusions:**

The reliability and the dimensionality of a condition-specific measure with high content validity for women having an abnormal cervical cancer screening results have been demonstrated. This new questionnaire called Consequences Of Screening in Cervical Cancer (COS-CC) covers in two parts the psychosocial experience in cervical cancer screening.

## Background

The purpose of cancer screening is to detect early stages of cancer and/or precursors hereof and thereby potentially decrease the incidence, the morbidity and/or the mortality of the cancer. These desired beneficial effects are inevitably followed by unintentional, harmful effects, e.g. detection of indolent pathological conditions defined as overdetection or overdiagnosis [1]. The overdiagnosis leads to overutilisation, overtreatment, labelling and thereby negative psychosocial consequences [2].

In cervical cancer screening (hereafter referred to as a cervical screening) the purpose is to detect precursors: cervical dysplasia and hereby potentially reduce the incidence, the morbidity and the mortality of cervical cancer [3]. However, when the cytological diagnosis of dysplasia is histologically confirmed there is still a high rate of spontaneous regression. A systematic review found that approximately 99% of mild dysplasia (CIN1), 95% of moderate dysplasia (CIN2) and 88% of severe dysplasia (CIN3) did not progress to cervical cancer [4]. Hence, cytological cervical screening will inevitably detect indolent dysplasia that leads to labelling, in some cases overtreatment and thereby can lead to negative psychosocial consequences.

Previous studies have shown that an abnormal cytological test can cause an increase in anxiety level and amount of distress [5–7], worries about infertility [6, 8, 9] and sexuality [6, 9, 10], and the perception of an increased risk of developing cancer [8, 9, 11]. Measurement of psychosocial consequences of cancer screening requires questionnaires with high content validity and adequate psychometric properties [12]. In a systematic review about the adequacy in measurement of psychosocial consequences in breast cancer screening the inadequacy of generic questionnaires has been revealed [13]. In another systematic review on psychological harm of screening it was concluded that the evidence on psychological harms is inadequate because of inadequacy in number of studies, in research design and measures [14]. We have previously developed two condition-specific questionnaires with high content validity and adequate psychometric properties to measure short and long term psychosocial consequences in breast cancer screening (the Consequence of Screening in Breast Cancer (COS-BC)) [15, 16] and in lung cancer screening (Consequence of Screening in Lung Cancer (COS-LC)) [17]. In our work, we found a common core-questionnaire COS (Consequence of Screening) for these two measures, i.e. the items and dimensions comprising the core-questionnaire COS have been shown to be relevant and valid in breast cancer screening and lung cancer screening. An unanswered question is if COS is also relevant in a setting of cervical screening. Therefore, the aim of the present study was threefold:

1. to examine the content relevance and content coverage of the core items of the COS in a setting of cervical screening;
2. if lack of content coverage of the COS was revealed, to generate themes and new items especially relevant for participants in cervical screening and to test the items for suitability;
3. if new items were generated, to test the extended version of the COS for dimensionality using Item Response Theory Rasch models.

## Methods

### Data collection: Content relevance and content coverage of the COS for application in cervical screening

Interviewees were recruited via Department of Pathology, Hvidovre Hospital (DoPHH) in May and June 2008 in order to test the relevance and content coverage of the COS for women with an abnormal cervical screening result.

When this study was carried out, triage tests among women aged 23–29 were not performed, and the Danish guidelines for women diagnosed with mild dysplasia (Atypical Squamous Cells of Undetermined Significance [ASCUS] & Low grade Squamous Intraepithelial Lesion [LSIL]) in this age range were cytological follow-up after 6 months, performed by general practitioners (GPs). For women of the age of 30 years or older diagnosed with mild dysplasia (ASCUS & LSIL) an HPV (human papillomavirus) test was performed. If the HPV test was negative the women were offered a cytological follow-up after 12 months, performed by GPs. If the HPV test was positive the women were referred to a gynaecologist for a pelvic examination including colposcopy and most often also cervical biopsies. Women diagnosed with severe dysplasia (High grade Squamous Intraepithelial Lesion [HSIL]) were referred to a gynaecologist for a pelvic examination including colposcopy and cervical biopsies no matter their age. In accordance to these different downstream procedures and to receive the greatest variation in information about what kind of psychosocial consequences women experienced after an abnormal cytological cervical screening test women were invited to group interviews strategically as listed in Table 1.

**Table 1:** Characteristics of the women invited to the group interviews

Group interviews	Abnormal screening test	Number of invited participants (age range)	Number of actual participants	Inclusion criteria
1	ASCUS & LSIL	20 (< 30 years)	4	Waiting for their 6-month follow-up
2	ASCUS & LSIL	20 (< 30 years)	1	1–12 months after a 6-month normal follow-up result
3	HSIL	20 (23–65 years)	2	After a normal colposcopy and cervical biopsies
4	ASCUS & LSIL + HPV negative	20 (> 30 years)	4	Waiting for their 12-month follow-up
5	ASCUS & LSIL + HPV positive or negative	20 (> 30 years)	6	1–12 months after a 12-month normal follow-up result

*ASCUS* Atypical Squamous Cells of Undetermined Significance, *LSIL* Low grade Squamous Intraepithelial Lesion, *HSIL* High grade Squamous Intraepithelial Lesion, *HPV* Human papillomavirus

The group interviews was planned to last approximately 2 hours consisting of two parts:

1. The first part as an open-ended discussion on the psychosocial consequences of abnormal and false-positive cervical screening results. The conceptualisation of ‘psychosocial consequences’ was based on the bio-psycho-social model in which people are not regarded as passive: they are considered able to both interact with and influence the environment [18].
2. in the second part the interviewees were asked to complete the COS and to comment on the relevance of the items.

All the COS-items are ordered thematically in Table 2.

**Table 2:** Content of the core-questionnaire COS (Consequence of Screening)

Themes or single items	The items of the COS. The number indicates the order of appearance in the questionnaire
Part I	
Anxiety	2. worried about my future
	3. scared
	12. upset
	13. restless
	14. nervous
	23. terrified
	25. shocked
Behavioural	4. irritable
	5. quieter than normal
	8. hard to concentrate
	10. change in appetite
	17. withdrawn into myself
	20. difficulty dealing work or other commitments
	22. difficulty doing things around the house
Sense of dejection	1. worried
	9. time passed slowly
	11. sad
	15. uneasy
	18. unable to cope
	19. depressed
Sleep	6. slept badly
	16. taken long time to fall asleep
	21. woken up far too early in the morning
	24. awake most of the night
Single items	7. busy to take mind off things
	71. less interest in sex
	33. sick leave
Part II	
Cervical cancer	3. anxiety about cervical cancer
	13. not cervical cancer
Relaxed/calm	4. relaxed
	8. calm
	17. relieved
Social relations	5. family
	6. friends
	7. other people
Existential values	1. broader aspects of life
	2. enjoyment of life
	9. thought about future
	10. well-being
	11.awareness of life
	12. value life
Impulsivity	14. energy
	16. lived life to the full
	19. being impulsive
	21. desire to venture into something new
	22. desire to venture into something risky
	23. done some things that overstepped one’s bound
Empathy	15. responsibility for one’s family
	18. understand other people’s problems
	20. ability to listen to other people’s problems

Part I of the COS encompasses three single items and four dimensions (including 24 items), in total 27 items with each four response categories: ‘not at all’, ‘a bit’, ‘quite a bit’ and ‘a lot’ [15–17]. If new items were generated in a group interview, the participants in the preceding group interviews would be asked to complete a draft to a new questionnaire called COS-CC (Consequences Of Screening in Cervical Cancer) that encompassed the items from the COS plus the new items specifically relevant for women in cervical screening.

Part II of the COS encompasses six dimensions including 23 items [15, 17]. The dimension “breast/lung cancer” encompassing two items in Part II was for obvious reasons renamed into ‘cervical cancer’. The response options in part II are five categorical variables on a continuum: ‘much less’, ‘less’, ‘the same as before’, ‘more’ and ‘much more’ ordered on two continuums. In previously conducted group interviews including informants who had false-positive results from screening mammography it was uncovered that the women’s experiences in the period from abnormal screening mammography until final false-positive diagnosis were completely different from their experiences after the final diagnosis [15]. In addition, the women argued that these completely different issues could only be raised after being declared ‘free from’ suspicion of cancer [15]. The informants reported these issues as long-term psychosocial consequences of false-positive screening mammography [15]. The women also argued that the consequences of the final diagnosis negative as well as positive consequences [15]. These findings were confirmed in five group interviews with men and women participating in a lung cancer screening trial [17].

It was planned that the participants of the first and fourth group interviews only should complete part I of the COS-CC because at the time of the interview the women had not been offered any follow-up of their abnormal screening results (Table 1). In group interviews number 2, 3 and 5 the participants were planned to complete versions of both parts of the COS-CC. In the group interviews, cognitive interviewing was also carried out item-by-item and included assessment of understandability, content relevance and content coverage [15]. Moreover, all the response options were reviewed for relevance and ease of completion.

In the COS-BC part II, each item includes the response option ‘no change’ indicating an anchor relative to two other options of changes in opposing directions. People’s preferences, values and perceptions of life can change as a result of existential crisis. Such changes can be positive, negative or a combination of both [17]. Therefore, Part II of the COS requires a special item scoring pattern because a traditional mean score of the dimensions does not reflect the actual distribution of changes. Rasch models presuppose that changes occur in only one direction. Therefore, any change from ‘The same as before’ should be regarded as long-term psychosocial consequences of screening. Thus, the responses to part II are ‘laterally reversed’ coded as: ‘much less’ or ‘much more’ is a variable of ‘much less/much more change’, ‘less’ or ‘more’ is a variable of ‘less/more change’ and finally ‘The same as before’ is a variable of ‘no change’ [17]. Rasch analyses on data collected with the COS-BC and COS-LC have confirmed these theories and assumptions [17, 19]. Moreover, the greatest fit to Rasch models have been achieved when using the ‘laterally reversed’ scoring of the response categories in part II [17, 19].

The test version of the questionnaire including any new items was planned subsequently to be field tested in single interviews among women from the group interviews. Easiness of completion and comprehension of the layout easy were tested in these single interviews.

The group interviews were audio-recorded and independently assessed by the JB and HT conducting thematic analyses to determine the key consequences of abnormal cervical screening results. These identified themes were discussed in detail in the following group interviews. In addition, the informants’ verbatim comments were used to develop and validate constructs, specifying a range of intensity from, for example, ‘little’ to ‘severe’ *negative experiences from the pelvic examination*. To avoid redundancy, items belonging to a construct were qualitatively compared pair-wise to ensure they did not have the same intensity. Finally, if JB’s and HT’s assessments did not correspond, the relevant sequences from the audio-recording were re-audited and discussed until consensus.

### Data collection for statistical psychometric analysis

Data were collected from March 2009 to December 2010 in a prospective matched cohort study. A randomised controlled trial (RCT) was conducted as a sub-study in the prospective matched cohort study with meditation as an intervention (ClinicalTrials.gov number, NCT00842738).

Participants were matched on date and place of analysis of the cytology test. Eligible were women who; were aged 23–29 years, had a cytology test taken by a GP and analysed in the DoPHH were never earlier diagnosed with cervical dysplasia, and could read and understand Danish. Exclusion criteria were: women with a known psychiatric diagnosis or dementia and women earlier diagnosed with cancer, apart from non-melanoma skin cancer.

Participants in the prospective matched cohort study consisted of an ASCUS/LSIL group (including women diagnosed with ASCUS or LSIL) and a control group (including women with a normal cytological test result). Participants in the RCT consisted of all participants from the ASCUS/LSIL group in the prospective matched cohort study.

Initially, women in the ASCUS/LSIL group were included in the project via GPs with residence in Copenhagen and the surrounding municipalities. Information regarding cytological test results was obtained from the DoPHH. After receiving information about the cytological test results, the principal investigator sent a letter to the woman’s GP, containing information about the study. The women’s GPs were asked to invite the women to participate in the study. It became clear for the project group rather quickly that this was a barrier for recruiting women, as the GPs did not always remember to ask if the patient wanted to participate.

Therefore, a new strategy for recruiting women was used; when a woman was diagnosed with ASCUS or LSIL at the DoPHH, the DoPHH sent an email to the principal investigator. The principal investigator then sent an invitation letter directly to the woman, and did not contact the woman’s GP. When a woman agreed to participate in the project, an email was sent to the DoPHH to find a woman with a normal cytological test result, analysed the same day as the women with ASCUS/LSIL (control group). An invitation letter was sent directly to the individuals of the control group. Three months after the primary cytological tests of the control group, the women were asked to complete the COS-CC.

Three months after the primary cytological tests of the ASCUS/LSIL group, the women were asked to complete part I of the COS-CC.

Seven months after the primary cytological tests of the ASCUS/LSIL group was taken, contact to DoPHH was made with the purpose of gaining information about the results of the ASCUS/LSIL group’s six-month follow-up cytology test. The results were collected and divided into two groups - “normal six-month follow-up cytology test” or “abnormal six-month follow-up cytology test” - through the DoPHH diagnosis-code. The former group included all women with a normal six-month follow-up cytology test, the latter included all women with a six-month follow-up cytology test diagnosis of one of the following: LSIL, ASCUS or HSIL. Participants of this group were by normal procedure referred to a gynaecologist for biopsy and histological diagnosis. Three months after the six-month follow-up cytology test both groups were asked to complete the COS-CC.

After the inclusion of all participants, and before being asked to complete the questionnaire, the women in the ASCUS/LSIL group were randomly allocated into two groups: a meditation group and a non-meditation group. When a woman agreed to participate in the study, she was randomised to one of the two groups using a randomisation-list generated at http://www.randomization.com/. The project-manager knew nothing else about the woman but her name, address, civil registration number and that the woman had been diagnosed with low-grade dysplasia. The project-manager was not blinded in relation to the randomisation-list. The principal investigator was blinded to the randomisation-list. The meditation group received a CD with four different mindfulness meditation exercises (breathing meditation, bodyscan, mountain meditation and sitting meditation) together with the first questionnaire. They were recommended to meditate twice a week during the study period. The women decided themselves when and which meditation exercise to use.

All women were sent the COS-CC by post and were asked to complete and return the questionnaire in an enclosed stamped addressed envelope. Those women who had not returned the questionnaire within 2 weeks were posted a reminder.

### Statistical analyses on dimensionality

Evaluations of the fit to the Rasch model were done in graphical log-linear Rasch models (GLLRM) [20]. These are a flexible class of models that imposes a conditional independence structure on the items, scale and exogenous variables, all assumed categorical; violations of the Rasch model are then identified as particular conditional independence hypotheses. Overall Rasch model fit and overall assessment of differential item functioning (DIF) was evaluated using Andersen’s conditional likelihood ratio test (CLR-χ^2^) [21]. By comparison of observed and expected correlations between scores for separate items and the summated *rest-scores* over all other items, individual item fit to the Rasch model was assessed by conditional infits and outfits [20]. Criterion validity and DIF were assessed by calculation of the degree of association between the item and exogenous variables conditional on the total scores using Goodman & Kruskal’s γ coefficient, as all variables are ordinal in response structure [22]. Exogenous covariates for DIF analysis were diagnostic group (normal screening result, abnormal screening result [ASCUS or LSIL], normal six-month follow-up cytology test and abnormal six-month follow-up cytology test [LSIL, ASCUS or HSIL]), time of assessment, age group, working status, living alone and social group. Local response dependency was assessed by the degree of association between two items conditional on the rest-score of one of them. The Benjamini-Hochberg procedure was used to account for multiple testing [23]. All analyses were conducted using DIGRAM [24].

Reliability was assessed by Cronbach’s alpha defining a lower bound for the test-retest correlation of the raw scores.

Items that present a misfit to the partial credit Rasch model (defined as statistically significant after a correction with the Benjamini-Hochberg procedure [25]), items possessing DIF, disordered thresholds, are regarded as ‘poor’ item because of their problematic measurement properties [17]. The measurement properties of scales encompassing one or more ‘poor’ items will be affected, e.g. if a ‘poor’ item has extensive DIF in a certain direction, then the data will suggest that DIF will operate in the opposite direction for other items in the scale: the DIF will level out for the remaining items on the scale. Therefore, an item possessing ‘real’ DIF can affect other items to show DIF; a DIF that is artificial. If an item possessing real DIF is split, then the fit to the Rasch model should increase – and vice versa if item split was conducted on an item possessing artificial DIF [17].

The plan for the Rasch analyses was the following: The items included in each theme in the COS and the items included in each new cervical screening-specific theme were analysed individually to test whether the items in a theme fitted the partial credit Rasch model. ‘Poor’ items revealing the greatest magnitude of psychometric ‘problems’ were deleted from the theme stepwise, except for for items possessing uniform DIF. Thereafter, a Rasch analysis was conducted including the remaining items composing the theme. If one or more items possessing uniform DIF were identified, all the items covering the theme were tested using GLLRM [20].

The item on sick leave (no. 33, Table 2) and the other single items (Tables 1 and 2) were not included in the Rasch analyses because these items did not belong to any of the dimensions.

## Results

### Results from the interviews

Altogether, 17 women participated in five group interviews and of those, eight women were interviewed in the period from an abnormal screening result until 6 or 12 month follow-up (Table 1).

Five women participated in the field test. During these field tests, only minor editing was conducted e.g. ‘more than usual’ was added to the item ‘I have been aware of my weight’, a phrase that was already included in several other items. Another example was that the word ‘other(s)’ had to be highlighted in items 32, 34 and 46 (see Table 1). No items were changed in part II.

The informants found all items in the COS relevant. In addition, ten themes specifically relevant for the critical period from abnormal cervical screening result until follow-up were extracted from the interviews: ‘Uncertainty about the screening result’, ‘Uncertainty about future pregnancy’, ‘Change in body perception’, ‘Change in perception of own age’, ‘Guilt’, ‘Fear and powerlessness’, ‘Negative experiences from the pelvic examination’, ‘Negative experiences from the examination’, ‘Emotional reactions’ and ‘Sexuality’ (Table 3). All ten themes were generated in the first group interview. Altogether, 50 new items for part I were generated, where 10 of the items were new single items (they did not belong to any themes: items 26–32, 47, 54 & 60, Table 4) and the remaining 40 new items’ subject matter described different nuances of the ten new themes (Tables 3 and 4). The themes and the subject matter for all 50 new items were generated in the first group interview and accepted in the following group interviews. Moreover, two single items about ‘Sick leave’ and ‘Self-rated health’ (items 33 & 34, Table 4) were included in the questionnaire.

**Table 3:** Fit statistics and the Cronbach’s alpha of the dimensions of the COS-CC

Dimensions (Number of items)	CLR-χ^2^	Degrees of freedom	P	Cronbach’s alpha
Part I				
Anxiety (7)	27.78	20	0.1147	0.829
Behavioural (7)	22.16	20	0.3318	0.818
Sense of dejection (6)	15.19	17	0.5817	0.817
Sleep (4)	8.95	10	0.5371	0.759
Uncertainty about the screening result (4)	14.66	11	0.1984	0.723
Uncertainty about the screening result (3) minus item 46	9.40	8	0.3094	0.765
Uncertainty about future pregnancy (3)	5.11	8	0.7456	0.811
Change in body perception (7)	37.64	20	0.0098	0.833
Change in body perception (6) minus item 44	24.15	17	0.1153	0.846
Change in body perception (5) minus item 44 & 57	5.20	14	0.9828	0.845
Change in perception of own age (2)	0.00	5	1.0	0.654
Guilt (3)	4.74	8	1.0	0.785
Fear and powerlessness (4)	8.17	11	0.6977	0.712
Negative experiences from the pelvic examination (7)	28.71	20	0.0936	0.880
Emotional reactions (4)	14.35	11	0.2144	0.776
Emotional reactions (3) minus item 62	2.38	6	0.8820	0.790
Emotional reactions (2) minus item 62 & 63	0.00	4	1.0	0.827
Sexuality (7)	26.54	20	0.1487	0.832
Part II				
Cervical cancer (2)	0.54	3	0.9104	0.299
Relaxed/calm (3)	0.64	4	0.9588	0.557
Social network (3)	5.45	3	0.1415	0.673
Existential values (6)	13.31	8	0.1016	0.835
Impulsivity (6)	5.02	11	0.9303	0.832
Empathy (3)	4.32	5	0.5049	0.731

CLR-χ^2^: Andersen’s conditional likelihood ratio test [20]

**Table 4:** Summary of result from the psychometric analyses of part 1 of the COS-CC

The items of part I of the COS-CC in order of appearance in the questionnaire	Subscales and misfit	Observed	Expected	Gamma sd	Probability of fit to the Rasch model	Item difficulty	Single or ‘poor’ item
1. worried	Dejection	0.654	0.723	0.036	0.05612	−1.12	
2. worried about my future	Anxiety	0.678	0.691	0.038	0.74067	−0.65	
3. scared	Anxiety	0.751	0.685	0.043	0.12738	0.05	
4. irritable	Behavioural	0.616	0.761	0.035	0.00004^b^	− 0.39	
5. quieter than normal	Behavioural	0.783	0.742	0.040	0.30285	0.32	
6. slept badly	Sleep	0.823	0.800	0.035	0.51521	−0.29	
7. busy to take mind off things	Single item	NA	NA	NA	NA	NA	single item
8. hard to concentrate	Behavioural	0.793	0.748	0.038	0.23658	0.08	
9. time passed slowly	Dejection	0.690	0.730	0.047	0.40140	0.06	
10. change in appetite	Behavioural	0.750	0.738	0.045	0.78002	0.07	
11. sad	Dejection	0.771	0.708	0.038	0.10405	−0.50	
12. restless	Anxiety	0.597	0.690	0.041	0.02456^a^	− 0.23	
13. nervous	Anxiety	0.572	0.678	0.048	0.02701^a^	0.50	
14. upset	Anxiety	0.758	0.686	0.040	0.06831	−0.29	
15. uneasy	Dejection	0.734	0.701	0.041	0.41964	0.12	
16. taken long time to fall asleep	Sleep	0.766	0.785	0.037	0.60991	0.09	
17. withdrawn into myself	Behavioural	0.765	0.733	0.045	0.47122	0.15	
18. unable to cope	Dejection	0.762	0.714	0.054	0.37715	0.73	
19. depressed	Dejection	0.721	0.704	0.054	0.76003	0.95	
20. difficulty dealing work or other commitments	Behavioural	0.780	0.731	0.046	0.29645	0.23	
21. woken up far too early in the morning	Sleep	0.712	0.777	0.039	0.09015	0.02	
22. difficulty doing things around the house	Behavioural	0.771	0.730	0.047	0.37679	0.26	
23. terrified	Anxiety	0.874	0.691	0.072	0.01101^a^	0.77	
24. awake most of the night	Sleep	0.910	0.764	0.057	0.01040^a^	1.37	
25. shocked	Anxiety	0.702	0.687	0.050	0.76485	0.34	
26. felt unsafe	Single item	NA	NA	NA	NA	NA	‘poor’ item
27. felt sorry for myself	Single item	NA	NA	NA	NA	NA	‘poor’ item
28. have considered going to my doctor	Single item	NA	NA	NA	NA	NA	‘poor’ item
29. thought one’s situation hopeless	Single item	NA	NA	NA	NA	NA	‘poor’ item
30. experienced mood swings	Single item	NA	NA	NA	NA	NA	‘poor’ item
31. more tired than usual	Single item	NA	NA	NA	NA	NA	‘poor’ item
32. keeping things from those who are close to you	Single item	NA	NA	NA	NA	NA	‘poor’ item
33. Sick leave	Single item	NA	NA	NA	NA	NA	single item
34. Self-rated health	Single item	NA	NA	NA	NA	NA	single item
35. the screening result has made me uncertain	Uncertainty about the screening result	0.714	0.657	0.045	0.20824		
36. as if there is something wrong with my body	Change in body perception	0.868	0.835	0.029	0.25147	−0.36	
37. experienced that I lost control	Fear and powerlessness	0.519	0.608	0.064	0.16434	1.07	
38. thought my body was vulnerable	Change in body perception	0.780	0.835	0.029	0.05896	−0.23	
39. my own fault	Guilt	0.839	0.761	0.045	0.08363	0.28	
40. aware of feeling something different in my lower abdomen	Single item	NA	NA	NA	NA	NA	‘poor’ item
41. afraid that I cannot get pregnant	Uncertainty about future pregnancy	0.873	0.844	0.026	0.25934	−0.75	
42. thoughts about the ability to become a mother	Uncertainty about future pregnancy	0.880	0.848	0.026	0.22174	−0.37	
43. as if it sits in my body	Change in body perception	0.819	0.832	0.031	0.67645	0.15	
44. felt older than my age	Misfit (Change in body perception)	0.521	0.747	0.045	< 0.000005^b^		
44. felt older than my age	Change in perception of own age	0.791	0.790	0.054	0.98406	−0.06	
45. unpleasant examination(s)	Negative experiences from the pelvic examination	0.828	0.785	0.029	0.14511	−0.60	
46. hard to trust that the screening result is true	Misfit (Uncertainty about the screening result)	0.341	0.544	0.057	0.00037^b^	NA	‘poor’ item
47. felt ill	Single item	NA	NA	NA	NA	NA	‘poor’ item
48. experienced that my body was a machine that does not work	Change in body perception	0.870	0.829	0.035	0.24821	0.56	
49. afraid that I would lose the baby if I got pregnant	Uncertainty about future pregnancy	0.760	0.837	0.036	0.02991	1.57	
50. felt sour (attitude)	Emotional reactions	0.906	0.906	0.029	0.99996		
51. angry	Emotional reactions	0.906	0.906	0.029	0.99996		
52. wondered if I should have taken better care of myself	Guilt	0.792	0.775	0.042	0.69616	−0.49	
53. Confused about what the screening result means	Uncertainty about the screening result	0.639	0.652	0.043	0.75952		
54. felt an emptiness	Single item	NA	NA	NA	NA	NA	‘poor’ item
55. Felt that my body was not my own body	Change in body perception	0.849	0.822	0.040	0.50265	0.93	
56. felt defenseless at the examination bed	Negative experiences from the pelvic examination	0.829	0.772	0.035	0.10738	0.38	
57. felt older than my age	Misfit (Change in body perception)	0.621	0.794	0.040	0.00002^b^		
57. felt older than my age	Change in perception of own age	0.791	0.790	0.054	0.98406	0.21	
58. felt vulnerable at the examination bed	Negative experiences from the pelvic examination	0.767	0.783	0.031	0.60707	0.00	
59. felt powerless	Fear and powerlessness	0.702	0.598	0.052	0.04647	0.66	
60. The idea that may be I am unable to have children has made me unhappy	Single item	NA	NA	NA	NA	NA	‘poor’ item
61. surprised that something was wrong	Uncertainty about the screening result	0.642	0.677	0.042	0.41331		
62. frightened	Emotional reactions	0.587	0.685	0.048	0.04082^a^	NA	‘poor’ item
63. cried more than usual	Emotional reactions	0.783	0.717	0.054	0.03860^a^	NA	‘poor’ item
64. felt humiliated at the examination bed	Negative experiences from the pelvic examination	0.772	0.768	0.038	0.92520	0.55	
65. felt that the examinations were painful	Negative experiences from the pelvic examination	0.776	0.776	0.034	0.99270	0.14	
66. felt that I had to overstepped my bounds at the examination bed	Negative experiences from the pelvic examination	0.779	0.778	0.033	0.97687	0.15	
67. felt I was unlucky	Fear and powerlessness	0.595	0.617	0.045	0.62384	−0.27	
68. fear of cervical cancer at the back of one’s mind	Fear and powerlessness	0.661	0.629	0.044	0.46986	−0.86	
69. been afraid to infect a partner	Guilt	0.590	0.719	0.064	0.04317	0.36	
70. felt that the examinations has been a tough experience	Negative experiences from the pelvic examination	0.658	0.770	0.042	0.00708^a^	0.61	
71. less interest in sex	Sexuality	0.764	0.768	0.045	0.92867	−0.37	
72. The idea of being with a partner again has been unpleasant	Sexuality	0.876	0.769	0.061	0.07887^a^	0.43	
73. had painful intercourse	Sexuality	0.716	0.774	0.048	0.22501	−0.23	
74. felt sting during intercourse	Sexuality	0.736	0.788	0.051	0.30588	−0.25	
75. had vaginal dryness	Sexuality	0.598	0.763	0.049	0.00075	0.06	
76. The idea of intercourse has been repulsive	Sexuality	0.846	0.764	0.067	0.21977	0.73	
77. negative impact on my sex life	Sexuality	0.925	0.780	0.049	0.00288^a^	− 0.13	

^a^Adjusting the *p*-values in the table in order to control the false discovery rate and so avoid spurious significant results due to multiple testing suggested that this result should be regarded as insignificant [23]

^b^Misfit after a correction of Benjamini-Hochberg procedure [23]

### Results of the data collection for the statistical psychometric analysis

At inclusion, 116 women diagnosed with LSIL or ASCUS accepted to participate in the RCT and 56 were allocated to the meditation group and 60 to the non-meditation group. Of these, 114 (98.3%) completed part I of the COS-CC. At the 3-month assessment time point after the women’s abnormal cytological screening result 75 (64.7%) of the 116 eligible women completed part I of the COS-CC. Three months after the six-month follow-up cytology test 63 (57.8%) of the 109 eligible women completed the COS-CC; seven women were not eligible because three had unknown address and four did not have any six-month follow-up cytology test. Of the 116 women with normal screening results matched to the group diagnosed with LSIL or ASCUS, 71 (62.3%) of the 114 eligible women completed the COS-CC three months after their primary normal cytological screening; one woman was not eligible due to unknown address and one was only 20 years old.

### Results from the Rasch analyses

#### Part I

##### Dimensionality of the core-questionnaire COS

All four dimensions fitted the partial credit Rasch model forming scales of: ‘Anxiety’, ‘Sense of dejection’, ‘Negative impact on behaviour’ and ‘Negative impact on sleep’ (Table 3). Item 4 ‘Irritable’ belonging to the ‘Negative impact on behaviour’ scale showed misfit to the model (Table 4) while at the same time the overall fit to the scale was very sufficient (Table 3). No DIF was revealed in any of the items in the four core-dimensions. Minor degrees of local dependence were revealed among some of the items in the dimensions ‘Anxiety’, ‘Negative impact on behaviour’ and ‘Negative impact on sleep’.

##### Dimensionality of the cervical screening-specific items

All items covering the themes: ‘Uncertainty about future pregnancy’, ‘Guilt’, ‘Fear and powerlessness’, ‘Negative experiences from the pelvic examination’, ‘Negative experiences from the examination’ and ‘Sexuality’ fitted the partial credit Rasch model (Tables 3 and 4). Minor degrees of local dependence were revealed among some of the items in the ‘Uncertainty about future pregnancy’, ‘Guilt’ scale, ‘Negative experiences from the pelvic examination’ and ‘Sexuality’ scales. Item 67 ‘Felt I was unlucky’ belonging to the ‘Fear and powerlessness’ scale possessed uniform DIF in relation to diagnosis group. None of the remaining items in the six scales: ‘Uncertainty about future pregnancy’, ‘Guilt’, ‘Fear and powerlessness’, ‘Negative experiences from the pelvic examination’, ‘Negative experiences from the examination’ and ‘Sexuality’ possessed DIF to any of the covariates.

Item 46 ‘hard to trust that the screening result is true’ a priori thought to belong to the ‘Uncertainty about the screening result’ scale possessed uniform DIF in relation to time plus revealed misfit to the Rasch model (Table 4). Cronbach’s alpha and the overall fit of the scale ‘Uncertainty about the screening result’ increased after deleting item 46 (Table 3). Thereafter, none of the remaining three items in the ‘Uncertainty about the screening result’ scale possessed DIF or had local dependency (Table 4).

Item 62 ‘frightened’ in the ‘Emotional reactions’ scale possessed uniform DIF in relation to time and diagnosis plus revealed marginal fit to the Rasch model (Table 4). After deleting item 62 from the ‘Emotional reactions’ scale, the overall fit to the model increased. However, item 63 ‘cried more than usual’ possessed uniform DIF in relation to time, diagnosis and age group, and revealed poor fit to the Rasch model (Table 4). After deleting both item 62 and 63 from the ‘Emotional reactions’ scale the two remaining items: 50 ‘felt sour’ and 51’angry’ fitted the partial credit Rasch and none of the items possessed DIF.

From the group interviews it was revealed that seven items described different nuances of the theme ‘Change in body perception’: items 36, 38, 43, 44, 48, 55 & 57 (Table 5). Two of these items (items 44 & 57, Table 5) did more specifically describe a theme the women called ‘Change in perception of own age’. In the Rasch analyses it was confirmed that the items 44 & 57 did not fit with the other five items in scale about ‘Change in body perception’ by showing misfit to the Rasch model (Tables 3 and 4). Therefore, the items were analysed in two separate scales. Items 36, 38, 43, 48 & 55 fitted the Rasch model forming a ‘Change in body perception’ scale (Tables 3 and 4), where none of the five items had local dependency but 43 possessed uniform DIF in relation to diagnosis. Items 44 & 57 fitted the Rasch model forming a ‘Change in perception of own age’ scale (Tables 3 and 4), where none of the two items had local dependency but both items possessed uniform DIF in relation to time and diagnosis.

**Table 5:** Summary of result from the psychometric analyses of part 2 of the COS-CC

The items of part I of the COS-LC in order of appearance in the questionnaire	Subscales and misfit	Observed	Expected	Gamma Sd	Item difficulty	Probability of fit to the Rasch model
1. broader aspects of life	Existential values	0.731	0.830	0.055	−0.66	0.07071
2. enjoyment of life	Existential values	0.819	0.836	0.074	14.02	0.82062
3. anxiety about cervical cancer	Cervical cancer	0.266	0.270	0.139	0.44	0.97745
4. relaxed	Relaxed/calm	0.562	0.525	0.118	0.23	0.74978
5. family	Social relations	0.778	0.849	0.066	−1.13	0.16247
6. friends	Social relations	0.947	0.802	0.061	−0.38	0.07890
7. other people	Social relations	0.918	0.881	0.062	1.51	0.51973
8. calm	Relaxed/calm	0.504	0.453	0.148	−0.04	0.72928
9. thought about future	Existential values	0.815	0.834	0.071	14.02	0.79353
10. well-being	Existential values	0.811	0.834	0.071	14.02	0.75552
11. awareness of life	Existential values	0.862	0.821	0.058	0.25	0.48193
12. value life	Existential values	0.941	0.824	0.060	0.42	0.05019
13. not cervical cancer	Cervical cancer	0.266	0.270	0.139	−0.44	0.97745
14. energy	Impulsivity	0.768	0.869	0.065	−0.55	0.11894
15. responsibility for one’s family	Empathy	0.799	0.867	0.071	0.33	0.33944
16. lived life to the full	Impulsivity	0.845	0.855	0.068	0.40	0.87765
17. relieved	Relaxed/calm	0.439	0.518	0.116	−0.21	0.49453
18. understand other people’s problems	Empathy	0.912	0.880	0.060	−0.51	0.59919
19. being impulsive	Impulsivity	0.918	0.858	0.069	−0.02	0.38448
20. ability to listen to other people’s problems	Empathy	0.880	0.870	0.065	0.18	0.88236
21. desire to venture into something new	Impulsivity	0.918	0.866	0.058	0.08	0.37147
22. desire to venture into something risky	Impulsivity	0.847	0.855	0.071	0.46	0.91448
23. done some things that overstepped one’s bounds	Impulsivity	0.856	0.862	0.065	−0.37	0.92278

Adjusting the *p*-values in the table in order to control the false discovery rate and so avoid spurious significant results due to multiple testing suggested that this result should be regarded as insignificant [23]

#### Part II

##### Dimensionality of part II of the core-questionnaire COS

All items in the six dimensions fitted the partial credit Rasch model regarding the overall all fit statistics (Table 3) and the item fit statistics (Table 5). Both items in the ‘Cervical cancer’ scale possessed uniform DIF in relation to diagnosis. None of the items in the remaining five scales possessed DIF. Of all 23 items in the six scales in part II there was only minor local dependency between two items: item 19 ‘being impulsive’ and item 21 ‘desire to venture into something new’ in the impulsivity scale.

All the items’ thresholds were in order in all the Rasch analyses.

## Discussion

The four core-questionnaire COS scales in part I: ‘Anxiety’, ‘Sense of dejection’, ‘Negative impact on behaviour’ and ‘Negative impact on sleep’ were all found qualitatively relevant and psychometrically valid for women having abnormal and normal findings in screening for cervical cancer. This was also valid for the six dimensions from part II in COS: ‘Cervical cancer’, ‘Relaxed/calm’, ‘Social network’, ‘Existential values’, ‘Impulsivity’, and ‘Empathy’.

Concerning scales specifically relevant for women participating in cervical screening, ten new scales were developed: ‘Uncertainty about the screening result’, ‘Uncertainty about future pregnancy’, ‘Change in body perception’, ‘Change in perception of own age’, ‘Guilt’, ‘Fear and powerlessness’, ‘Negative experiences from the pelvic examination’, ‘Negative experiences from the examination’, ‘Emotional reactions’ and ‘Sexuality’. All ten dimensions were confirmed to measure different constructs: seven of the dimensions fitted a partial credit Rasch model, while three dimensions encompassed one or two items possessing DIF.

No new single items or dimensions for part II about the long-term psychosocial consequences were developed since content validity of this part of the COS was assessed high among the interviewees in the five group interviews.

A limitation of the present study is that for each group interview 20 women were invited but only a minor part of the invited wanted to participate in an interview. However, data saturation was already achieved in the first group interview and no new items or themes were generated in the following four group interviews. Therefore, it seems that the spectrum of psychosocial consequences of cervical screening might be the same no matter the downstream procedures followed by an abnormal screening result, which might not be the case about the severity of the psychosocial consequences and how long the women experience these consequences.

The present study revealed that having an abnormal screening result, later confirmed to be false-positive in breast and lung cancer screening and having an abnormal cervical screening result has something in common: the core-questionnaire COS has now been found to be relevant for those participants in all three screening programmes. Moreover, the ten scales in the COS: ‘Anxiety’, ‘Sense of dejection’, ‘Negative impact on behaviour’, ‘Negative impact on sleep’, ‘Cervical cancer’, ‘Relaxed/calm’, ‘Social network’, ‘Existential values’, ‘Impulsivity’, and ‘Empathy’ have all in each of the three settings be shown to fit Rasch models [16, 17, 19]. A future project would be to analyse if the ten scales also across the screening programmes measure the ten constructs invariantly.

Item 4 ‘Irritable’ belonging to the ‘Negative impact on behaviour’ scale showed misfit to the partial credit Rasch model despite the scale showing overall fit to the model. There could not be revealed any explanations to this item misfit. Therefore, it is too premature to delete this item from the scale since this item in a setting of breast and lung cancer screening has previously shown adequate psychometric properties [16, 17].

Ten new scales specifically relevant for women having abnormal cervical screening were developed and found unidimensional in Rasch models. However, seven items in six of the scales were identified as possessing uniform DIF. There was no obvious explanation to this DIF. Five of the seven items possessed DIF in relation to time. This is very problematic in a longitudinal design where repeated measurement is conducted [26]. Therefore, the items 44, 46, 57, 62 and 63 were all regarded as poor items and deleted from their respective scales. This also meant that the two-item scale ‘Change in perception of own age’, encompassing the items 44 and 57, had inadequate psychometric properties in a longitudinal design and therefore this scale cannot be used if repeated measurement is conducted. The items 43 and 67: ‘as if it sits in my body’ and ‘felt I was unlucky’ belonging to the scales ‘Change in body perception’ and ‘Fear and powerlessness’ respectively, possessed both uniform DIF in relation to diagnosis. Therefore, these items can be used without adjustment in a study where only women with abnormal or normal cervical screening are included [27], e.g. the abovementioned RCT where it is investigated if mindfulness meditation can lower the negative psychosocial consequences of having an abnormal cervical screening result. However, if comparison is done across diagnostic groups, adjustment has to be done in accordance to the magnitude of the uniform DIF [27]. Alternatively, the two scales ‘Change in body perception’ and ‘Fear and powerlessness’ could be used without item 43 and item 67 respectively.

In the present study, the approach used to develop and validate the Consequences Of Screening in Cervical Cancer (COS-CC) questionnaire: a condition-specific psychosocial measure for cervical screening, has revealed that when a qualitative content validity driven approach is used it is possible to develop a multi-dimensional measure with high content validity and adequate psychometric properties. In previous studies on measurement of psychosocial and/or quality of life aspect a more data driven approach was used [28–30]. Comparing the content of the COS-CC with these three previous developed measures reveals that the old measures lack content validity. An explanation to the differences could be the data driven approach: either because the development of the measures was not based on qualitative interviews with the target population and/or because the statistical psychometric analyses were not confirmatory but more exploratory. In addition, none of the old measures were validated using item response theory modelling.

## Conclusion

A new condition-specific questionnaire for women having an abnormal cervical screening result with high content validity was developed. This measure called Consequences of Screening in Cervical Cancer (COS-CC) covers in two parts the psychosocial experiences in cervical screening. Adequate reliability, uni-dimensionality and invariant measurement of the scales encompassed in the COS-CC have been demonstrated using Rasch models.

Part I: ‘Anxiety’, ‘Behaviour’, ‘Dejection’, ‘Sleep’, ‘Uncertainty about future pregnancy’, ‘Uncertainty about future pregnancy’, ‘Change in body perception’, ‘Guilt’, ‘Fear and powerlessness’, ‘Negative experiences from the pelvic examination’, ‘Negative experiences from the examination’ ‘Emotional reactions’ and ‘Sexuality’.

Part II: ‘Cervical cancer’, ‘Relaxed/calm’, ‘Social network’, ‘Existential values’, ‘Impulsivity’, and ‘Empathy’.

## Abbreviations

ASCUS: Atypical Squamous Cells of Undetermined Significance
CIN1: mild dysplasia
CIN2: moderate dysplasia
CIN3: severe dysplasia
CLR-χ^2^: Andersen’s conditional likelihood ratio test
COS: Consequence of Screening
COS-BC: Consequence of Screening in Breast Cancer
COS-CC: Consequences Of Screening in Cervical Cancer
COS-LC: Consequence of Screening in Lung Cancer
DIF: differential item functioning
DoPHH: Department of Pathology, Hvidovre Hospital
GLLRM: graphical log-linear Rasch models
GP: general practitioner
HPV: human papillomavirus
HSIL: High grade Squamous Intraepithelial Lesion
LSIL: Low grade Squamous Intraepithelial Lesion
RCT: randomised controlled trial
